# Assessing Dietary Habits, Quality, and Nutritional Composition of Workplace Lunches: A Comprehensive Analysis in Turin, Piedmont (Italy)

**DOI:** 10.3390/nu17162625

**Published:** 2025-08-13

**Authors:** Carla Ferraris, Walter Martelli, Aitor Garcia-Vozmediano, Maria Ines Crescio, Cristiana Maurella, Eleonora Mingolla, Elisabetta Fea, Andrea Pezzana, Paola Chiara Durelli, Lucia Decastelli, Daniela Manila Bianchi

**Affiliations:** 1 Centro di Referenza Nazionale per la Rilevazione negli Alimenti di Sostanze e Prodotti che Provocano Allergie e Intolleranze—CReNaRiA, Via Bologna 148, 10154 Turin, Italy; carla.ferraris@izsplv.it (C.F.); lucia.decastelli@outlook.it (L.D.); manila.bianchi@izsplv.it (D.M.B.); 2 Istituto Zooprofilattico Sperimentale del Piemonte Liguria e Valle d’Aosta, Via Bologna 148, 10154 Turin, Italy; aitor.garciavozmediano@izsplv.it (A.G.-V.); mariaines.crescio@izsplv.it (M.I.C.); cristiana.maurella@izsplv.it (C.M.); 3 Dipartimento di Scienze della Sanità Pubblica e Pediatriche, Università degli Studi di Torino, Via Santena 5 bis, 10126 Turin, Italy; eleonora.mingolla@edu.unito.it (E.M.); elisabetta.fea@unito.it (E.F.); 4 SC Nutrizione Clinica, ASL Città di Torino, Via Silvio Pellico 28, 10128 Turin, Italy; andrea.pezzana@aslcittaditorino.it (A.P.); paolachiara.durelli@aslcittaditorino.it (P.C.D.)

**Keywords:** nutritional quality of lunch, healthy eating plate, dietary habits

## Abstract

**Background:** The importance of understanding dietary habits during working hours is becoming increasingly evident. As demonstrated, dietary habits have been shown to exert a considerable influence on the productivity of workers and the creation of a healthier workplace. **Objective**: The aim of this study is to assess the nutritional quality and self-perception of lunches consumed by workers in Piedmont (Italy). **Methods:** A questionnaire, supported by the EasyDietWeb 4.3.0 software, was used to assess the macronutrient composition of the meals and to evaluate adherence to the “Healthy Eating Plate” (HEP) guidelines. The basal metabolic rate and total daily energy expenditure of the subjects were calculated. **Results:** The survey results, which included 189 participants, revealed that a notable proportion of the respondents consumed homemade meals at their place of work. The majority of meals reported by the participants did not align with the HEP composition due to the absence of one or more components, especially vegetables. The participants’ perceptions of meal balance frequently diverged from the actual nutritional quality of the meals. Finally, overweight participants exhibited a higher risk of reporting diseases (OR = 4.4, 95% CI = 1.6–12.0). **Conclusions:** This study provides insight into the dietary habits of a specific group of workers regarding their lunch consumption. This underscores the significance of enhancing public awareness regarding dietary choices and nutritional intakes, as adhering to proper dietary routines is paramount for preserving a state of well-being and sustaining a healthy lifestyle.

## 1. Introduction

Recent decades have seen a number of societal changes that have had a notable impact on human behaviour and lifestyles, including dietary habits [1]. Globalisation, changes in time use, and improvements in transport are among the main factors that have led to a global increase in sedentary behaviour and away-from-home food intake [2]. According to the Statistical Office of the European Union, household expenditure on catering services increased across the EU member states between the years 2008 and 2018 [3]. The increasing necessity of consuming meals outside the home, coupled with the demand for time-saving dietary solutions, has led to a significant uptake of ready-to-eat (RTE) foods that can be consumed without any additional preparation [4]. The changes in dietary patterns exert a considerable influence on the selection of meals consumed during working hours. According to Jaworowska et al. (2018), a large percentage of workers (approximately 70%) opt for external dining options, with around 30% of these depending on on-site canteens for purchasing their meals [5]. Given that a significant portion of an adult’s time is allocated to professional activities, the food environment in workplaces should be considered a factor to include in health assessments [5]. Several studies have explored the role of workplace eating habits as a crucial factor in maintaining workers’ productivity [6,7]. Nutritional health issues have been associated with a decline in productivity and overall well-being, with balanced and correct diet showing a positive influence on work and mental performance [8]. Conversely, sedentary behaviour, which is often observed in office environments, contributes to the development of obesity, overnutrition, and diseases such as diabetes or metabolic syndrome, in addition to an unbalanced diet [8,9,10]. The creation of a healthy food environment and adequate and proper nutrition are important aspects of a healthy lifestyle [11,12] A recent intervention in a work canteen, aimed at promoting healthy and sustainable food choices, has yielded a positive effect on the nutritional composition of meals [13]. Despite the growing recognition of the role of nutrition in overall health and well-being, there are still few studies that have examined the accessibility of food, particularly in workplaces and in public places [12], which sometimes do not have a canteen, meaning that people have to bring food from home or buy it. It is therefore essential to understand dietary habits in the workplace to facilitate the development of effective health promotion initiatives grounded in nutrition [13]. In this context, the objective of this study is to evaluate the lunch-related eating habits, the quality, and the nutritional composition of lunches consumed at work by the staff of the Istituto Zooprofilattico Sperimentale del Piemonte, Liguria e Valle d’Aosta (IZSPLV). Furthermore, the lifestyle of the participants was evaluated in terms of physical activity; also, lunch-related eating habits were evaluated in relation to the presence or absence of pathological conditions, as well as their perceptions of the consumption of balanced meals at work.

## 2. Materials and Methods

### 2.1. Survey Design, Data Collection, and Parameter Estimation—Nutritional Profile Assessment of Meals

We designed an anonymous online questionnaire using Google Forms, titled “What Did You Have for Lunch Today?”, to conduct a qualitative and quantitative assessment of the meals consumed in the IZSPLV workplace as a convenience sample (Supplementary Material S8: Questionnaire). In the questionnaire, respondents were invited to provide a recall of their lunch on the previous day at the workplace. Typically, the lunch interval in our institution occurs between 12:00 p.m. and 02:00 p.m. Consequently, any repast during the lunch break was categorised as “lunch”. This approach aimed to capture a snapshot of eating behaviours in the workplace.

The questionnaire was structured in three main sections, with the objective of evaluating (i) the respondents’ health status and the dietary habits observed at lunchtime, (ii) the quality and the nutritional composition of the meals consumed at the workplace, and (iii) the respondents’ beliefs about the balance of their meals (Supplementary Materials). Most of the questions incorporated closed multiple-choice answers, thereby enabling categorical classification for the purpose of analysis. Foodstuffs such as crackers, condiments, oil, and butter were appraised by quantity categories (e.g., number of packets or tablespoons). Quantitative variables pertaining to body weight, height, and number of eggs consumed were entered directly by the participants. Open-text fields were used to indicate the presence of any medical conditions. As the IZSPLV is not equipped with its own canteen or cafeteria, but instead has one lunchroom with two microwaves, a further question addressed the provenance of the lunch, with the response options encompassing home-prepared meals or food items purchased from gastronomy establishments, restaurants, and cafeterias situated near the workplace. Such establishments usually provide lunch between 12:00 p.m. and 02:00 p.m., with a selection of both ready-to-eat food, such as toast and salads, and hot entrées comprising dishes such as pasta, rice, meat, vegetables, and fish.

To ensure the respondents’ objectivity, questions referring to quantitative assessments of food were accompanied by pictures from the Photo Food Atlas by the Scotti-Bassani Institute [14], a reference manual for dietitians on food portions and quantities that is widely used for the estimation of portion size [15,16,17]. The following food portions were indicated: small (40–50 g), medium (70–80 g), and large (120–150 g).

The survey was conducted on two separate days in November 2021 (11th and 16th November), using a convenience sampling strategy and targeting the personnel of the IZSPLV. The survey was distributed to participants via email invitation. The questionnaire was administered to the IZSPLV personnel on two occasions, thus enabling the acquisition of a larger sample for analysis. The IZSPLV is engaged in the conduct of controls and research in food safety and animal health, as well as the performance of epidemiological investigations in the field of nutrition. Thus, we wanted to investigate whether the diet in the workplace was actually nutritionally adequate.

Portion size assessment

The stated portions (in grams) obtained from the respondents’ self-reported answers in the questionnaire were then compared with those outlined in the fourth edition of the Italian standard servings [18] in order to assess the potential differences in intake levels. The following factors were taken into account to achieve this: self-reported sex, age group, body mass index (BMI), the origin of the meal, and whether the meal was considered a balanced one. The food items consumed in the meal by the various participants were classified according to the food groups outlined in the Dietary Reference Intake for the Italian Population (LARNs) [18].

Assessment of the energy content of meals

The energy content (expressed in kcal) and macronutrient composition (expressed in grams and percentages) of the meals were calculated using the EasyDietWeb software (version 4.3.0), a dietary assessment tool commonly employed by dietitians and other professionals for the nutritional analysis of meals. The software includes a comprehensive database of food items, accompanied by their respective nutritional profiles. In instances where the food consumed at lunchtime was not included in the EasyDietWeb 4.3.0 database, the nutritional values were sourced from the CREA Food Composition Database, a popular and online Italian database containing the nutrient composition of foods. The results obtained were then compared with the standard portion sizes described by the Italian Society of Nutrition [18]. In addition, the results were classified according to the nutritional partitioning delineated by the Department of Nutrition of Harvard T.H. Chan School of Public Health in the Healthy Eating Plates (HEP) [19]. In particular, meals were deemed to be in accordance with the HEP if they comprised a portion of vegetables, a portion of carbohydrates, a portion of proteins, and a portion of lipids. Fruit can be consumed either during the meal or as a standalone snack [20], so it was not included in this evaluation. Finally, to ascertain whether the lunch was calorically adequate, a range of 25–35% was calculated for each total daily energy expenditure (TDEE), in accordance with the recommendations set forth in the Italian Dietary Guidelines.

### 2.2. Estimation of Basal Metabolic Rate and Total Daily Energy Expenditure

To estimate the basal metabolic rate (BMR) and the total daily energy expenditure (TDEE), data on subjects’ self-reported age, sex, body weight, and height were requested. Information regarding the physical activity of the participants was collected based on the type of work they performed (classified into three categories: sedentary, moderately active, and active work) and their physical activity outside of work (divided into six ordinal categories, ranging from no activity to four days per week). To predict BMR, the equation of Schofield et al. (1985) was used (Table S2) [21]. TDEE was instead calculated by multiplying the BMR by the physical activity level (PAL), in accordance with the recommendations set out by the UK Scientific Advisory Committee on Nutrition (SACN) [22] and the Italian Dietary Guidelines [13]. PAL is a factor for quantifying the amount of physical activity, as formulated by the FAO/WHO/UNU expert committee in 1985 [16]. Specifically, we estimated PAL through the combination of self-reported physical activity (categorised into six levels, as mentioned above) and self-reported type of work (Table S3). In particular, the 2018 Healthy Eating Guidelines for Italians recommend that adults should engage in “at least 150 min of moderate physical activity or at least 75 min of vigorous physical activity each week, or an equivalent combination of both”. Additionally, muscle-strengthening activities are also recommended. Moreover, the BMI was calculated from self-reported height and weight data (body weight in kilograms divided by the square of height in metres) and was further categorised into four groups: underweight (BMI < 18.49 kg/m^2^), normal weight (BMI 18.5 to 24.99 kg/m^2^), overweight (BMI 25 to 29.9 kg/m^2^), and obese (BMI > 30 kg/m^2^). For the purposes of statistical analysis, the BMI was categorised into two distinct groups: those with a BMI in the normal weight range (BMI 18.5 to 24.99 kg/m^2^), and those with a BMI outside the normal weight range (BMI < 18.49 kg/m^2^; BMI > 25 kg/m^2^).

Supplementary Tables S5 and S6 show the Schofield equation and estimation of TDEE with PAL levels for calculating individual indicators.

### 2.3. Statistical Analysis

The collected data were managed and analysed using Stata software (Stata 18.5, College Station, TX, USA). After a descriptive statistical analysis, we evaluated the relationship between BMI, caloric intake in the meal, and balanced/unbalanced meal intake with respect to anthropometric characteristics and individual habits. The differences between the groups were assessed by means of Pearson’s chi-squared test for binary variables. To quantify the associations between groups, a series of univariate and multivariate analyses were carried out. Odds ratios (ORs) were calculated by means of logistic regression.

The Kruskal–Wallis test (KW test, a generalised form of the Mann–Whitney test, allowing for 2 or more groups) was used to analyse the relationships between the portion size of meals and sex, age group, BMI, meal origin, and balanced/unbalanced meals. For all analyses, a *p*-value lower than *p* = 0.05 was considered statistically significant.

## 3. Results

### 3.1. Characteristics, Habits, and Health Status of the Respondents

A total of 189 respondents completed the online questionnaire. The total number of potential respondents in November 2021 was 344 individuals.

The participants were predominantly female (77.2%), and over 40 years of age (74.6%). A comparable distribution in age was observed among male participants (Table S1). Most of the respondents generally self-reported good health and good physical condition, while a smaller proportion of participants declared being affected by some common health issues, including hypertension, hypercholesterolemia, or other diseases, as well as being underweight (4.9%) or overweight/obese (24%). We uncovered differences in BMI according to self-reported sex, with males demonstrating a higher propensity for overweight/obese condition compared to females (OR = 5.3, 95% CI = 2.5–11.2). These results were further supported by an age-adjusted linear regression between BMI (treated as a continuous variable) and sex, which revealed that, on average, BMI was 3.7 units (95% CI = 2.5–4.8) higher in males than in females.

With regard to the type of work conducted (sedentary, partially active, and active work), the respondents indicated that they were engaged in sedentary work in 64.5% of instances. Conversely, the respondents reported that they had active work in 33.3% of cases (with 2.12% of the respondents reporting partially active work). In relation to the physical activity undertaken during the course of the week, sedentarism was the most prevalent, with 53.4% of the respondents reporting no physical activity or poor physical activity (less than twice a week). The remaining respondents reported practising physical activity from one to more than three times per week (46.6%).

In terms of dietary habits, most of the respondents indicated that their diet was predominately omnivorous, and that their daily water consumption was less than two litres. Only 6.3% of participants reported following an alternative diet, which did not differ significantly based on age or sex (e.g., flexitarian, vegetarian, vegan, etc.). Additionally, 12.7% of respondents reported consuming more than two litres of water per day. No statistically significant differences were identified between the various dietary groups with regard to sex, the presence of underlying health conditions, meal perceptions, and BMI (Table S1). The consumption of homemade meals at the workplace was the prevalent dietary practise among the respondents (69.5% of participants).

The univariate analysis showed that the following categories were more likely to have a BMI outside the normal weight range: males (compared to females), participants reporting diseases (compared to those reporting good health status), participants aged between 30 and 39 years (compared to those aged 20–29 years), and those with a low caloric intake (compared to those with an adequate intake). Moreover, there was a tendency, which increased with both age and sedentary work, to purchase meals from restaurants. Finally, the presence of disease reported by participants increased with age (Table 1).

### 3.2. Nutritional Composition of Lunches

**Figure 1:**
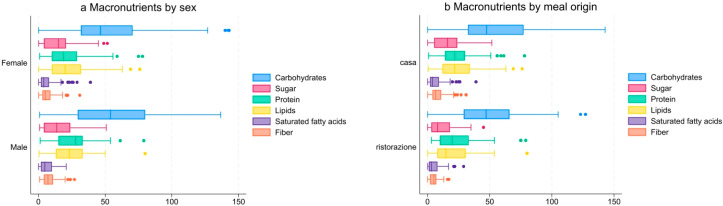
Box-and-whisker plots showing the intake (in grams) of nutrients by sex (**a**) and meal origin (**b**).

Meals at the workplace were characterised by high contents of carbohydrates, with a median value of 47 g. The intake of macronutrients was found to be generally similar between both sexes, except for protein intake, which was observed to be lower in females compared to males (averaging 25 g and 28.8 g, respectively; Table S7, Figure 1a). A comparison of home-prepared meals with those provided by restaurants revealed statistically significant disparities in the contents of sugar (*p* = 0.002) and fibre (*p* < 0.001), with higher concentrations observed in home-prepared meals. However, the Kruskal–Wallis test revealed no significant variations in the other nutrients examined (Table S7, Figure 1b).

From a qualitative perspective, the analysis of the respondents’ dietary habits revealed that the majority of individuals did not adhere to a balanced diet, as defined by the nutritional guidelines set forth by the HEP. Specifically, 66.7% of the respondents were found to consume unbalanced meals. The main cause that led to this conclusion was the absence of vegetables (51.6%), either in solo consumption or in combination with other macronutrients, such as proteins (23.4%) and carbohydrates (22.3%).

### 3.3. Evaluation of Portion Size

The portions obtained from self-reported answers were compared with those recommended by the Italian standard servings. Differences in the intake of food categories and the variables considered are illustrated in Table 2. Differences were identified for the intake of fats that equalled or exceeded the median intake, according to sex and meal origin. An odds ratio (OR) of 2.7 (95% CI = 1.3–5.3) was observed for females with respect to males, and an OR of 2.3 (95% CI = 1.2–4.4) was observed for home-prepared meals compared to food services. Further differences were found for above-median intake of the meat, fish, and eggs group when considering the BMI, with an OR of 1.8 (95% CI = 0.91–3.4) for respondents with higher BMI compared to those displaying normal BMI. Moreover, above-median fruit intake was found to be associated with the origin of the meal, with an OR of 2.5 (95% CI = 1.3–4.6) for home-prepared meals compared to food services, and with participants assuming balanced diets (OR = 2.1, 95% CI = 1.1–4.0). Finally, differences in the above-median consumption of vegetables were notable when evaluating the meals as balanced or not balanced. The OR for this outcome was 10.1 (95% CI = 5.1–20.6), indicating an increased likelihood of vegetable consumption for participants who consumed a balanced meal compared to those who did not.

### 3.4. Evaluation of the Energy Content of the Meals

Of the 189 respondents, 5 female subjects were excluded from this analysis because they did not provide data regarding their body weight, thus preventing the calculation of both BMR and TDEE. The total number of respondents included in the evaluation of the energy content of meals was 184.

**Figure 2:**
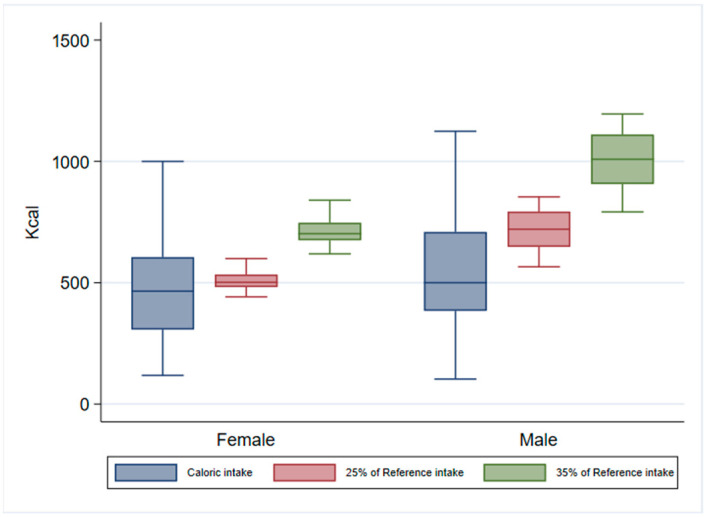
Caloric intake and values recommended by the Italian Dietary Guidelines (25–35% of TDEE) in both sexes.

To investigate whether the caloric intake provided by the meal was in accordance with the Italian Dietary Guidelines (25–35% of TDEE), we compared the caloric intake of each individual meal with the TDEE of the respondents. It was observed that, on average, the intake in kcal was lower than the reference intake values of 25% and 35% of TDEE for the meal in question. The mean caloric intake was 479.3 kcal in females and 543.2 kcal in males (Table S4). By comparing these data by sex, the caloric intake was lower than the values of 25% and 35% taken as references for the meal (Figure 2). By categorising the caloric intake according to the Italian Guideline reference intake, we observed that this parameter was low in 47% of meals, adequate in 46%, and high in 7%. The likelihood of observing a caloric intake different from the recommended values was higher among participants consuming unbalanced meals (OR = 2.1, 95% CI = 1.1–4.0). Tables S5a,b and S6a,b show the BMR, PAL, TDEE, and 25–35% of TDEE of the 184 respondents.

### 3.5. Self-Perception of Diet

**Figure 3:**
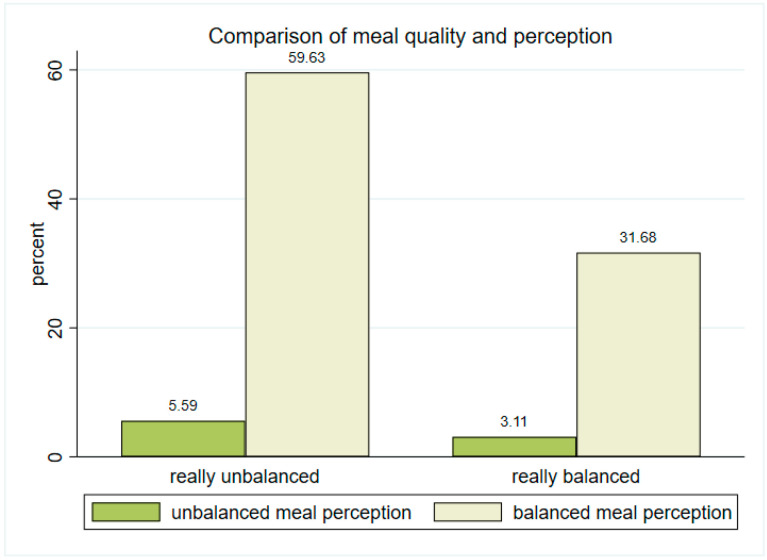
Comparison of meal quality with the perceptions of respondents. Note: The graph does not include respondents who did not state whether they consider their diet to be balanced or not.

Based on the data obtained regarding the balanced or unbalanced intake of macronutrients and calories, we observed that 72.5% of the respondents perceived their meals to be balanced (described as “a diet consisting of a variety of all the different types of food and providing adequate amounts of the macronutrient”). By comparing HEP-based balanced meal data against participants’ perceptions of meal balance, we found that 59.6% of the respondents who believed that they were eating a balanced meal did not actually meet the HEP standard. Conversely, 31.7% of individuals who self-reported adherence to a balanced diet exhibited a balanced macronutrient profile in their actual dietary intake (Figure 3). The chi-squared test was not significant (*p* = 0.939) in assessing the association between a balanced diet and the respondents’ perceptions. Regarding the comparison between the indicated diet and the actual caloric intake obtained from the meal, it was observed that 48.2% of the respondents who indicated having a balanced meal had a low caloric intake, while only 5.6% of this same group had a high caloric intake. In 46.1% of cases, the caloric intake was adequate when a balanced diet was indicated.

## 4. Discussion

This study provides a snapshot of the working lunch habits of employees at the IZSPLV in Piedmont, Italy, including assessments of meal quality and self-perception. The aim of this study was not to generalise to the broader population or to examine seasonality dietary variations, but rather to capture a single moment in time.

This study yielded several noteworthy findings. Firstly, individuals’ perceptions of their meals often did not correspond to the actual caloric content and macronutrient distribution. Secondly, purchasing meals from restaurants was associated with respondents of older age and with sedentary behaviour. Thirdly, although the probability of deviating from the recommended caloric intake was higher among participants consuming unbalanced meals, a balanced meal did not necessarily equate to an appropriate caloric intake.

Studies examining workplace food quality in Italy remain scarce [13,23]. In 2020, the CIRFOOD Good Lunch Break Observatory conducted a survey of 1200 adults, including students and workers, aged 18–55 [24]. The results from this survey revealed that most workers dined out two to three times per week, approximately 65% opted for home-packed meals, and 21% utilised food delivery services. Our finding of a 69.5% preference for home-prepared meals aligns closely with this range.

We evaluated associations among sex, BMI, physical activity, meal origin, and meal energy and macronutrient contents. Male respondents were more likely to have a BMI outside the normal range, despite similar types of work or physical activity profiles, suggesting that additional factors may contribute to the higher BMI in men. This finding aligns with those of studies indicating a higher prevalence of overweight among men compared to women in high-income countries, including Italy, possibly reflecting the influence of sociocultural factors [25]. Furthermore, we observed that older adult participants exhibited a greater propensity to consume meals purchased at restaurants, contrasting with the findings of other studies reporting higher out-of-home consumption among younger adults [26,27].

Another interesting finding is that most of the individuals in our study were engaged in sedentary work with low levels of physical activity, which is consistent with patterns observed in similar professional contexts [28,29]. Given the predominantly laboratory- and office-based environment of our institute, these findings underscore the importance of dietary quality for health maintenance.

Our data highlight the challenge of achieving a balanced macronutrient and caloric intake in a professional setting. Office meals frequently failed to comply with the HEP composition guidelines and often provided inadequate energy levels. As a single meal per respondent was captured, our results are insufficient to infer overall dietary balance throughout the day. Therefore, the results should be interpreted as an assessment of workplace meal adequacy rather than total daily intake.

The predominant macronutrient in workplace lunches was carbohydrates, consistent with the Mediterranean dietary principles, which advocate a carbohydrate intake of up to 60% of TDEE [30]. However, the observed intakes of protein and fibre were both below the LARNs recommendations. For fibre intake, for example, we observed that the amount consumed during work meals was lower than the recommended reference daily amounts of fibre (>25 g/day) [18]. This might suggest that without additional fruits, vegetables, or wholegrain foods outside their workplace meal, the participants are unlikely to meet their daily fibre targets. Although concentrated exclusively on workplace lunches, our findings illustrate the need for greater emphasis on balanced meal provision at work. A recent systematic review confirmed the effectiveness of interventions promoting healthy meals away from home and underlined the importance of consumer education [31].

Portion size analysis indicated that individuals with a higher BMI consumed greater amounts of meat, fish, and eggs. Women and participants bringing homemade lunches consumed higher levels of fat. The highest levels of fruit consumption were also found in participants who bring food from home. These findings partially align with previous research linking red meat consumption to obesity [32] and vegetable intake with lower adiposity [33], and they support evidence that home-cooked meals tend to be healthier than those eaten out [34,35,36]. To the best of our knowledge, a few studies have directly compared portion sizes with guideline recommendations. Some studies have shown that the provision of portion information can support healthier choices, thus contributing to overall well-being [37,38]. Moreover, frequent home cooking has been associated with normal-range BMI [39], whereas reliance on meals prepared away from home has been shown to be related with inadequate fibre and fruit intake [40] and increased adiposity [41].

Furthermore, we identified an incongruity between individuals’ perceptions of meal balance and the actual caloric and macronutrient composition. This finding stands in contrast to the results of a previous study, which indicated that the participants exhibited good dietary awareness [42]. This suggests a potential lack of awareness concerning the nutritional and caloric contents of foods among the study participants. In this survey, we did not directly assess nutritional literacy; a previous study demonstrated a strong association between nutritional knowledge and adherence to healthy dietary patterns, particularly the Mediterranean diet [43]. In addition, female subjects were predominant in our sample, which is consistent with the higher propensity of women to participate in surveys, as shown in other studies [44,45], and reflects the sex distribution of our workforce [40,46,47].

Although the single-meal surveys resemble the 24 h recall method, they are subject to similar limitations. Factors such as the day of the week, the mode of interview (i.e., telephone, face to face, online survey), and the number of recalls can influence reported energy intakes. In fact, this method has the potential to capture a wider variety of foods, as it refers to a meal made recently. In contrast, seasonal variability, for example, can introduce a bias in the estimates of food and nutrient intakes [48]. The reliability of self-reported dietary data is questionable due to the potential for underreporting, and underestimation of true BMI is a common occurrence [49] due to weight underreporting and height overestimation [50,51,52]. Nevertheless, self-reported and measured anthropometric measures generally demonstrate high concordance.

We acknowledge that our study has several limitations that should be considered when interpreting the findings. Firstly, the sample consisted solely of personnel from the IZSPLV institute, limiting the generalisability of the results to the general population. Secondly, we focused on a single meal per respondent, precluding an assessment of total daily dietary intake and the potential for improving nutritional behaviours throughout the day. Additionally, we did not include a direct evaluation of the participants’ nutritional knowledge, limiting our ability to explore the influence of nutritional literacy on meal choices. Finally, seasonal dietary variation was not assessed, as our data collection focused on a single point in time, which could affect the representativeness of food choices.

## 5. Conclusions

Through this survey, we attempted to assess the dietary habits of a sample of personnel employed at the IZSPLV, in the workplace, evaluating meal quality against HEP composition and LARNs portion guidelines. To the best of our knowledge, this is one of the few studies to integrate all of these aspects (i.e., macronutrient contents, portion sizes, and self-perception) in a single analysis. Our results have practical implications. The observed discrepancy between the perceived and actual healthiness of meals emphasises the necessity for targeted nutritional education in the workplace. Interventions could include the provision of informational workshops and easily accessible educational materials, such as leaflets in canteens, bars, restaurants, and cafeterias, detailing HEP meal composition and portion guidance. Employers can promote healthier dietary habits and contribute to the well-being of their workforce by raising awareness of meal quality and promoting balanced choices.

## Figures and Tables

**Table 1:** Odds ratios (ORs) and 95% confidence intervals (95% CIs) from univariate logistic regression models assessing the influence of demographic and lifestyle factors on (1) BMI (reference = outside the normal range), and (2) meal origin (reference = restaurant) and health status (reference = disease).

BMI (Case BMI = Outside Normal Weight Range)			
		OR	95% CI
Sex	Female	Ref	
	Male	4.3	2.1–8.7
Health Status	Good health	Ref	
	Disease	2.1	1.1–4.1
Age group	20–29	Ref	
	30–39	4.5	1.2–17.0
	40–49	1.35	0.40–4.5
	>49	1.94	0.6–6.5
Caloric intake	Poor	2.6	1.3–5.3
	Adequate	Ref	
	In excess	0.8	0.2–4.2
Meal Origin (Case = Restaurant)			
Age group *	20–29	Ref	
	30–39	0.4	0.03–4.9
	40–49	4.7	1.0–21.0
	>49	8.8	1.9–40.9
Type of work	Active	Ref	
	Sedentary	2.5	1.2–5.1
Health Status (Case = Disease)			
Age group *	20–29	Ref	
	30–39	5	0.5–46.5
	40–49	7.7	1.0–61.2
	>49	16.8	2.1–132.7

The multivariate analysis showed that overweight participants exhibited a higher risk of reporting disease (OR = 4.4, 95% CI = 1.6–12.0). This association was also observed among participants over 40 years of age (40–49 years: OR = 9.8, 95% CI = 1.14–84.4; >49 years: OR = 27.0, 95% CI = 3.0–245.0). * Significative score test for trend of odds.

**Table 2:** Mean (standard deviation) and median (iqr) of food categories’ intake by sex, age group, BMI category, meal origin, and balanced meal.

Groups		n	Dairy Products (g)	Meat, Fish, Eggs (g)	Legumes (g)	Cereals and Derivatives Tubers (g)	Vegetables (g)	Fats Condiments (g)	Fruit (g)
			Mean (sd) Median (iqr)	Mean (sd) Median (iqr)	Mean (sd) Median (iqr)	Mean (sd) Median (iqr)	Mean (sd) Median (iqr)	Mean (sd) Median (iqr)	Mean (sd) Median (iqr)
Sex	Female	146 (77.2%)	44.5 (77.3) 0.0 (125.0)	44.1 (57.3) 0.0 (80.0)	11.0 (40.7) 0.0 (0.0)	48.9 (46.1) 35.0 (70.0)	44.9 (74.3) 0.0 (60.0)	**7.4 (7.9)** 5.0 (10.0) *	110.9 (116.8) 50.0 (150.0)
	Male	43 (22.8%)	33.3 (51.5) 0.0 (50.0)	55.6 (73.5) 20.0 (80.0)	20.0 (68.4) 0.0 (0.0)	57.4 (63.4) 50.0 (70.0)	30.7 (71.7) 0.0 (40.0)	**5.1 (8.3)** 0.0 (10.0) *	119.2 (134.1) 150.0 (150.0)
Age group	20–29	22 (11.6%)	35.5 (59.3) 0.0 (70.0)	49.8 (74.0) 0.0 (80.0)	0.0 (0.0) 0.0 (0.0)	49.1 (66.1) 30.0 (70.0)	31.8 (74.9) 0.0 (0.0)	6.4 (9.7) 5.0 (10.0)	157.5 (139.6) 150.0 (187.5)
	30–39	26 (13.8%)	28.1 (63.6) 0.0 (0.0)	39.6 (54.9) 0.0 (110.0)	18.8 (67.3) 0.0 (0.0)	62.1 (64.5) 45.0 (65.0)	62.9 (104.5) 0.0 (105.0)	9.8 (10.7) 10.0 (10.0)	118.8 (116.9) 150.0 (150.0)
	40–49	78 (41.3%)	39.5 (72.6) 0.0 (60.0)	55.3 (65.8) 40.0 (80.0)	7.8 (29.8) 0.0 (0.0)	47.5 (44.5) 40.0 (70.0)	35.2 (61.5) 0.0 (50.0)	5.9 (7.2) 5.0 (10.0)	89.4 (110.2) 0.0 (150.0)
	>49	63 (33.3%)	52.8 (78.7) 0.0 (125.0)	37.6 (52.2) 0.0 (80.0)	22.1 (63.9) 0.0 (0.0)	51.1 (45.4) 30.0 (40.0)	44.9 (73.0) 0.0 (60.0)	7.3 (7.1) 10.0 (10.0)	123.8 (124.1) 150.0 (150.0)
BMI	Underweight	9 (4.8%)	62.5 (115.7) 0.0 (125.0)	**0.0 (0.0)** 0.0 (0.0) **	0.0 (0.0) 0.0 (0.0)	58.8 (42.2) 55.0 (55.0)	56.2 (77.6) 0.0 (150.0)	3.1 (4.6) 0.0 (7.5)	112.5 (174.7) 0.0 (225.0)
	Normal	134 (70.9%)	44.4 (75.3) 0.0 (125.0)	**40.9 (54.4)** 0.0 (80.0) **	13.1 (47.7) 0.0 (0.0)	49.6 (48.6) 30.0 (70.0)	49.0 (81.3) 0.0 (60.0)	7.3 (8.5) 5.0 (10.0)	112.2 (116.8) 150.0 (150.0)
	Overweight	46 (24.3%)	30.8 (50.2) 0.0 (50.0)	**71.9 (75.9)** 80.0 (120.0) **	15.3 (54.5) 0.0 (0.0)	52.9 (57.7) 40.0 (70.0)	17.5 (36.4) 0.0 (20.0)	6.6 (6.9) 5.0 (10.0)	114.3 (123.1) 150.0 (150.0)
Balanced meal	Yes	62 (33.0%)	44.1 (74.6) 0.0 (70.0)	48.2 (52.6) 40.0 (80.0)	10.0 (33.4) 0.0 (0.0)	43.7 (35.5) 30.0 (20.0)	**82.7 (91.8)** 60.0 (150.0) ***	7.5 (8.3) 10.0 (10.0)	**142.0 (125.2)** 150.0 (225.0) *
	No	126 (67.0%)	40.2 (71.2) 0.0 (70.0)	45.7 (65.5) 0.0 (80.0)	13.4 (53.0) 0.0 (0.0)	53.7 (55.9) 40.0 (70.0)	**22.2 (53.7)** 0.0 (0.0) ***	6.6 (7.9) 5.0 (10.0)	**98.8 (116.7)** 75.0 (150.0) *
Meal origin	Home	130 (69.5%)	42.7 (72.6) 0.0 (125.0)	44.1 (55.3) 0.0 (80.0)	16.7 (55.5) 0.0 (0.0)	48.1 (50.2) 30.0 (70.0)	41.5 (70.8) 0.0 (60.0)	**7.9 (8.3)** 10.0 (10.0) **	**128.2 (118.2)** 150.0 (150.0) **
	Restaurant	57 (30.5%)	36.9 (71.3) 0.0 (20.0)	51.2 (74.6) 0.0 (80.0)	4.5 (23.1) 0.0 (0.0)	56.0 (49.0) 40.0 (80.0)	41.0 (81.2) 0.0 (55.0)	**4.1 (6.4)** 0.0 (10.0) **	**76.5 (120.5)** 0.0 (150.0) **

Groups in bold are statistically different, with (*) *p*-value < 0.05, (**) *p*-value < 0.01, and (***) *p*-value < 0.001 (Kruskal–Wallis equality of population rank test).

## Data Availability

Data are contained within the article.

## Supplementary Materials

The following supporting information can be downloaded at https://www.mdpi.com/article/10.3390/nu17162625/s1: Supplementary Material S8: Questionnaire. Table S1: Characteristics, habits, and health status of the respondents. Table S2: Schofield equation to predict BMR. BW = Body weight. Table S3: Estimation of TDEE based on self-reported type of work and physical activity. Table S4: Mean and median of TDEE, 25% and 35% of TDEE. Table S5a: Male, age 18–29: basal metabolic rate (BMR), physical activity level (PAL), total daily energy expenditure (TDEE), and 25–35% of TDEE. Table S5b: Male, age 30–60: basal metabolic rate (BMR), physical activity level (PAL), total daily energy expenditure (TDEE), and 25–35% of TDEE. Table S6a: Female, age 18–29: basal metabolic rate (BMR), physical activity level (PAL), total daily energy expenditure (TDEE), and 25–35% of TDEE. Table S6b: Female, age 30–60: basal metabolic rate (BMR), physical activity level (PAL), total daily energy expenditure (TDEE), and 25–35% of TDEE. Table S7: Nutrient intake for both sexes and meal origins. Sd = standard deviation; iqr = interquartile range (Kw: Kruskal–Wallis test).
