# Supplementary material for: Assessing Dietary Habits, Quality, and Nutritional Composition of Workplace Lunches: A Comprehensive Analysis in Turin, Piedmont (Italy)
Source: Nutrients. 2025 Aug 13;17(16):2625. doi: 10.3390/nu17162625 (PMC12389728; doi:10.3390/nu17162625)
Supplement: Supplementary file 1 [file nutrients-17-02625-s001.zip › Supplementary mat s8_nutrients.pdf]

Supplementary Materials

Questionnaire

WHAT DID YOU HAVE FOR LUNCH TODAY?
A Research Project of IZSPLV

The compilation is anonymous and voluntary, it will take no more than 10 minutes.

Your data will be used for scientific purposes, in compliance with the General Data Protection Regulation (Reg. EU 679/2016).

1. Your age group
   a. 20-29
   b. 30-39
   c. 40-49
   d. >50

2. I would describe my gender as
   a. Male
   b. Female
   c. I prefer not to respond

3. State your body weight (kilograms) and height (meters)
   a. height
   b. body weight

4. Indicate any medical conditions present (e.g., high cholesterol, diabetes, elevated triglycerides, high/low blood pressure, thyroid problems...)

5. Where was your lunch cooked?
   a. At home, homemade food
   b. Purchased ready-made at the gastronomy / restaurant /cafés
   c. Purchased ready-made at the supermarket

6. What did you have for lunch today? Choose from the following options:

First courses (pasta, rice, oats, barley, spelt, cous cous, quinoa and other grains...)

smaller	Like this	Like this	Like this	Bigger

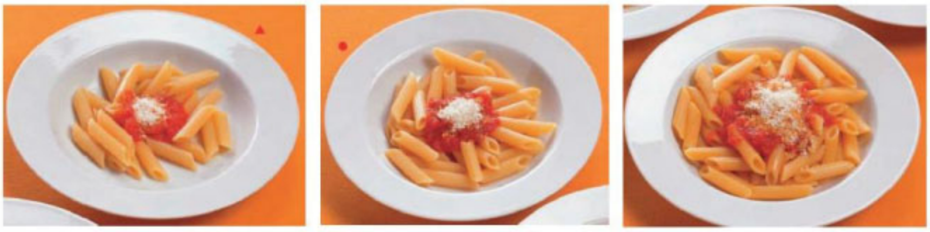

Was it whole wheat?

Was homemade pasta (like tagliatelle) or tortellini or gnocchi?

Pizza

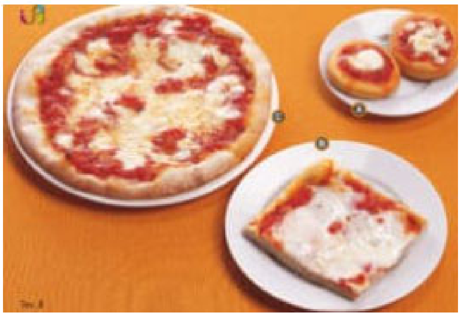

Meat and Fish

smaller	Like this	Like this	Like this	Bigger

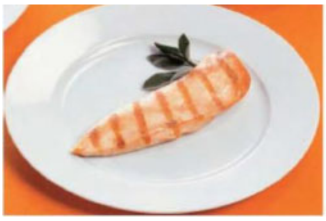
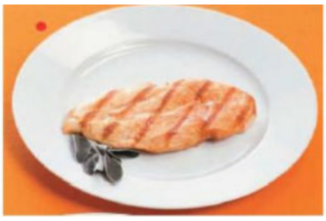
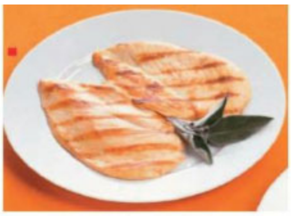

Canned meat or fish: indicate what and how much (e.g., 1 can of natural tuna, 2 cans of jellied meat...)

Legumes (chickpeas, beans of all kinds, lentils, peas, broad beans, lupins, soybeans)

smaller	Like this	Like this	Like this	Bigger

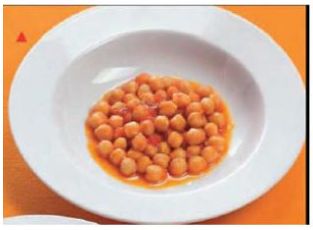
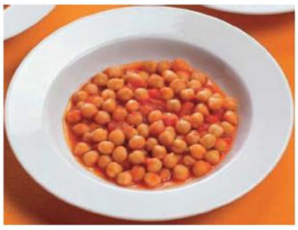
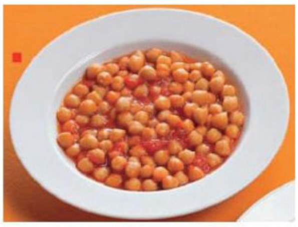

Hard and semi-hard cheeses (such as grana and parmesan, feta, pecorino, gorgonzola, toma, , emmental, philadelphia, buffalo mozzarella, scamorza...)

smaller	Like this	Like this	Like this	Bigger

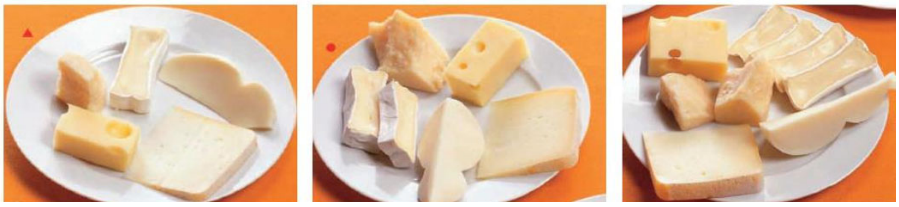

Soft cheeses (such as cottage cheese, cottage cheese, light mozzarella, light philadelphia...)

**smaller**	**Like this**	**Like this**	**Like this**	**Bigger**

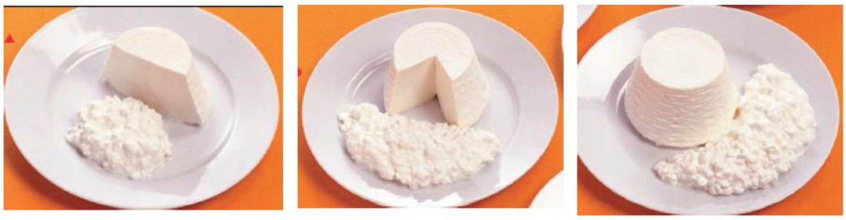

Uncooked salad

**smaller**	**Like this**	**Like this**	**Like this**	**Bigger**

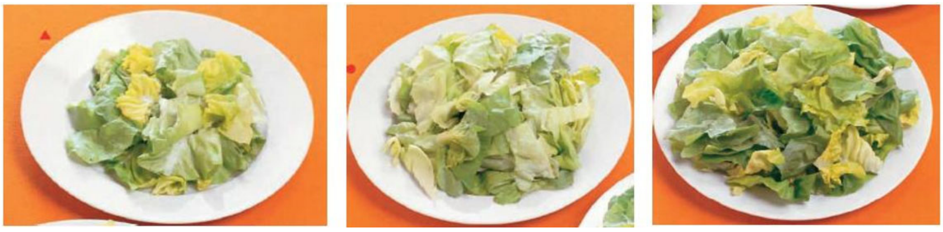

Minestrone soup

**smaller**	**Like this**	**Like this**	**Like this**	**Bigger**

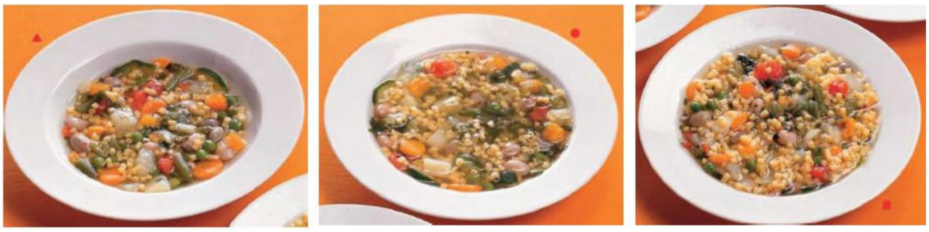

Fruit

a. Less than 1 fruit (e.g., half banana, half apple)
b. 1 fruit
c. 2 fruits
d. More than 2 fruits

Bread

**smaller**	**Like this**	**Bigger**

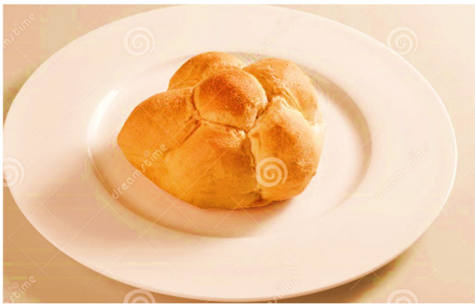

Was it whole wheat?

Crackers (e.g., corn crackers, rice crackers...) indicates the number
Crackers

a. Less than 1 packet
b. 1 packet
c. 2 packets
d. More than 2 packets

Was it whole wheat?

Ready-sliced meat

**smaller**	**Like this**	**Like this**	**Like this**	**Bigger**

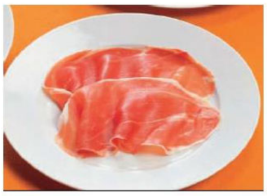
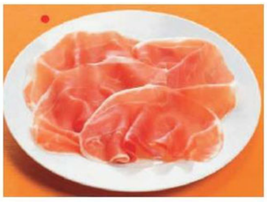
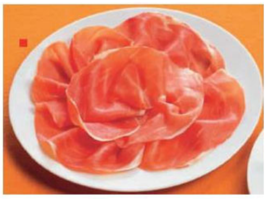

Eggs (number)

a. 1
b. 2
c. 3
d. 4

Potatoes

**smaller**	**Like this**	**Like this**	**Like this**	**Bigger**

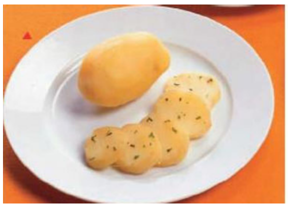
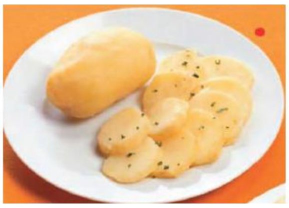
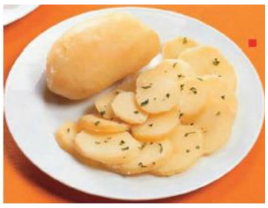

Yoghurt and similar products

a. Natural white yoghurt (or kefir)
b. Fruit yoghurt (or kefir)
c. Greek or skyr natural white yoghurt
d. Greek or skyr fruit yoghurt

Other foodstuffs e.g. SWEETS, DRIED FRUIT, SALTED CAKES, PIZZA, BAKERY PRODUCTS (specify)

7. How much oil did you use for cooking today?
   a. less than 1 tablespoon
   b. 1 tablespoon
   c. 2 tablespoons
   d. 3 tablespoons
   e. Don't know
   f. I don't use oil

8. How much butter did you use for cooking today?
   a. less than 1 tablespoon
   b. 1 tablespoon
   c. 2 tablespoons
   d. 3 tablespoons
   e. Don't know
   f. I don't use butter

9. How many condiments such as tomato sauce and pesto did you use for cooking today?
   a. less than 1 tablespoon
   b. 1 tablespoon
   c. 2 tablespoons
   d. 3 tablespoons
   e. Don't know
   f. I don't use condiments

10. How much water do you drink per day?
    a. less than 1 liter
    b. 1 liter - 1.5 liters
    c. 1.5 liters - 2
    d. More than 2 liters

11. Do you practice physical activity? Indicate the sport and how many times a week
    a. No physical activity
    b. Poor (less than 1 time/week)
    c. 1 time/week
    d. 2 times/week
    e. 3 times/week
    f. More than 3 times/week

12. How do you consider your personal diet?
    a. Varied and balanced (e.g., I eat a little bit of everything without excess)
    b. Boring and repetitive (Give an example)
    c. I follow a diet I don't like (Give an example)
    d. Unbalanced (e.g., I completely avoid a class of foods-such as vegetables, fruits, carbohydrates such as bread, pasta, rice, pizza, various cereals)

13. What kind of diet do you follow?
    a. Omnivorous
    b. Vegetarian
    c. Vegan

e. Other (specify)

14. Describe your work:
    a. Sedentary work: I spend most of my time sitting down
    b. Standing work: I spend most of my time standing or walking, but without doing intense physical exertion
    c. Manual work: some physical exertion is required, such as carrying heavy packages
    d. Other (specify)
